# Plant Exosome-like Nanovesicles and Their Role in the Innovative Delivery of RNA Therapeutics

**DOI:** 10.3390/biomedicines11071806

**Published:** 2023-06-24

**Authors:** Yu-Xin Chen, Qiang Cai

**Affiliations:** 1 Key Laboratory of Fermentation Engineering (Ministry of Education), Cooperative Innovation Center of Industrial Fermentation (Ministry of Education & Hubei Province), School of Biological Engineering and Food, Hubei University of Technology, Wuhan 430068, China; yuxinc@hbut.edu.cn; 2 State Key Laboratory of Hybrid Rice, Hubei Hongshan Laboratory, College of Life Sciences, Wuhan University, Wuhan 430072, China

**Keywords:** exosomes, plant exosome-like nanovesicles (PENVs), artificial PENV-derived nano-vectors (APNVs), small RNA delivery, nanomedicine

## Abstract

Exosomes are single membrane-bound spheres released from cells carrying complex cargoes, including lipids, proteins, and nucleic acids. Exosomes transfer specific cargoes from donor to acceptor cells, playing important roles in cell-to-cell communication. Current studies have reported that plant exosomes are prominent in transferring small RNA between host and pathogens in a cross-kingdom manner. Plant exosomes are excellent RNA interference (RNAi) delivery agents with similar physical and chemical properties to mammalian exosomes and have potential applications in therapeutic delivery systems. Recent data have suggested that plant exosome-like nanovesicles (PENVs) and artificial PENV-derived nano-vectors (APNVs) are beneficial for delivering therapeutic small RNA in mammalian systems and exhibit excellent competitiveness in future clinical applications. This review features their preparation methods, composition, roles in small RNA delivery for health functionalities, and their potency as functional nanomedicine.

## 1. Introduction

Exosomes are small extracellular vesicles (EVs) that originate from the fusion of the endocytic organelles, the multivesicular bodies (MVBs), with the plasma membrane [1,2]. Tetraspanins, such as CD9, CD63, and CD81 on the vesicle surface, are the most common exosome marker proteins [1,3,4]. The membrane of exosomes contains cholesterol, phosphatidylserine, sphingomyelin, and ceramides, which play an important role in cell-to-cell signaling and the stability of exosomes [5]. Exosomes also carry and transport genetic material (messenger RNAs (mRNAs), microRNAs (miRNA), and other non-coding RNAs) and proteins between cells, altering the physiological and pathological functions of recipient cells [1,6,7,8,9,10]. The cell origin of exosomes differs from the other two classes of EVs, microvesicles and apoptotic cell-derived EVs. Microvesicles normally directly bud from the plasma membrane and carry the surface membrane materials and cytoplasmic content of cells [11,12]. Apoptotic cell-derived EVs are released as a product of apoptotic cells undergoing programmed cell death [13,14,15]. Recent data have suggested that EVs have emerged as a major pathway to delivering functional molecules between cells and organisms that play important roles in immune response, antigen presentation, and cancer cell migration [1,16,17]. Nowadays, the application research on EVs in drug delivery, nutrition, clinical diagnosis, and treatment tends to be mature [9,18,19].

In plants, the term “plant EVs” is used for vesicles isolated from extracellular spaces, such as apoplastic wash fluid and cell culture media [20]. Nowadays, plant EVs have been isolated from apoplastic wash fluid of Arabidopsis and *Nicotiana benthamiana* leaves [21,22,23,24] and sunflower seeds [25]. Interestingly, a kind of plant EV, pollensomes, has been isolated from the germination media of in vitro-growing olive pollen tubes [26,27]. The latest studies showed that the expression of two CD63 homologs in Arabidopsis, *TET8* and *TET9*, were induced by fungal pathogen infection [23]. TET8 interacted with TET9 to form a protein complex enriched in the membrane of TET8-positive EVs. These EVs function as a defense system by delivering small RNAs into pathogens, mediating cross-species and cross-kingdom RNA interference (RNAi) to suppress fungal virulence genes [23,28,29]. During the infection, TET8-labeled organelles partially colocalize with the MVB marker Rab5-like GTPase ARA6 and accumulate at fungal infection sites, suggesting TET8-positive EVs derived from MVBs can be considered as plant exosomes [22,23]. Thus, plant exosomes function as a defense system by encasing and delivering bioactive molecules into pathogens, contributing to host immunity [20,30].

Like animal EVs, plant EVs consist of heterogeneous vesicles of different sizes and intracellular origins [31]. Another plant EV subtype, penetration (PEN)1-positive EVs, enriches a plasma membrane-associated syntaxin PEN 1 [24]. PEN1 does not colocalize with MVB marker ARA6 and TET8 inside the cell, indicating PEN1-positive EVs have distinct biogenesis pathways with TET8-positive EVs [21,22]. The EXPO is a novel plant organelle with a double-layer membrane structure [32,33]. After the outer membrane of EXPO fuses with the plasma membrane, the internal vesicle is released into the extracellular space as an EV [32,33]. Interestingly, EXPOs-derived EVs are large EVs whose diameter ranges from 200 to 500 nm, larger than exosomes (50–150 nm) [32,33]. The specific functions of PEN 1-positive EVs and EXPOs-derived EVs, and whether they contribute to delivering bioactive molecules between cells, are still unknown. The latest study showed the release of autophagy-related EVs was induced when Arabidopsis was infected by *Pseudomonas syringae* pv. *tomato* (*Pst*) DC3000 (*AvrRpm1*) [34]. These EVs are enriched with the autophagy marker ATG8a and may transport monolignol to the cell wall to inhibit bacterial growth [34].

Plant exosome-like nanovesicles (PENVs) are plant-derived nano-sized vesicles (50–1000 nm) with bioactivities, which are increasingly being studied for therapeutic drug delivery [35,36]. PENVs are not plant EVs, because they are obtained from plant tissues after disruptive processes. Because they were isolated from homogenized plant tissues, PENVs had higher extraction rates than plant EVs [37,38,39,40]. Indeed, PENVs contain a complex mixture of intracellular vesicles and EVs, and have physical characteristics like mammal EVs, including morphology, particle size, concentration, and zeta potential [41,42]. Several native biomaterials in PENVs have been identified and shown to have anti-inflammatory, anticancer, and tissue regenerative activities [40,43]. To further elucidate the specific molecular mechanisms of PENVs to maintain health or treat diseases, many studies have focused on the effective functional substances carried by PENVs, including lipids, proteins, nucleic acids, and other metabolites [36,44]. Nucleic acids in PENVs, especially small RNAs, can enter mammalian cells and mediate trans-kingdom gene regulation [45]. The membrane of PENVs contains certain lipids that can be assembled into artificial PENV-derived nano-vectors (APNVs) [46]. Therefore, APNVs can be packaged with active ingredients, such as small interfering RNAs (siRNAs), for various drug delivery. PENVs and APNVs act as small RNA and other bioactive molecule carriers, showing low immunogenicity, high efficiency, safety, and are highly economical, potentially impacting many human diseases [35,44]. 

## 2. Isolation and Purification for Plant EVs

**Figure 1:**
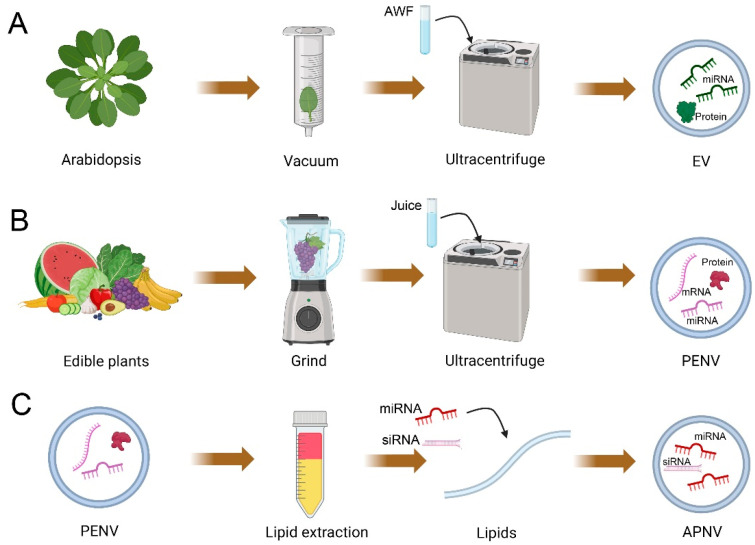
Schematic of the isolation of plant extracellular vesicles (EVs), artificial plant exosome-like nanovesicles (PENVs), and artificial PENV-derived nano-vectors (APNVs). (**A**) Images show EVs isolated from apoplastic washing fluid (AWF) using ultracentrifugation. (**B**) Images show PENVs isolated from plant juice using ultracentrifugation. Plant juice is obtained by dissociating the plant tissue in a blender (tissue-disruption method). (**C**) Images show that lipids of PENVs can be assembled into artificial PENV-derived nano-vectors (APNVs). This figure was created with BioRender.com.

Animal EVs are isolated from several biological fluids, while plant EVs are isolated from apoplastic wash fluid [21,47]. Based on the infiltration–centrifugation method, apoplastic wash fluid is commonly extracted from plant leaves by tissue infiltration and low-speed centrifugation to preserve cell integrity [21,48] (Figure 1). The detached leaves protocol is a superior choice for collecting apoplastic wash fluid to reduce damaged cells and contamination [21,49]. In this protocol, detached leaves are gently vacuumed with infiltration buffer within a large needleless syringe (100 mL syringe) and then are taped to a small needleless syringe (1 mL syringe) [21,49]. The wrapped syringe with taped leaves is placed into a conical tube and then centrifuged at low speed (900× *g*) to collect the apoplastic wash fluid [21,49]. The ideal contamination-free apoplastic wash fluid should be clear and transparent [21]. Like animal EV isolation, differential centrifugation is the most popular and widely used method for plant EV isolation from apoplastic wash fluid [20,21]. In this method, 2 consecutive steps of centrifugation at 2000× *g* and 10,000× *g* are used to remove dead cells, cell debris, and large vesicles. The supernatant is then centrifuged at 100,000× *g* to pellet small plant EVs [21,49]. Differential centrifugation is a widely accepted method that has been used to isolate EVs from different plant species, such as Arabidopsis leaves [22,23,24,34], *N. benthamiana* leaves [22], sunflower seeds [25], and olive pollen tubes [26,27]. 

Specific parameters, such as centrifugal forces, affect plant EV compositions. EVs final centrifuged at 100,000× *g* (P100) or 40,000× *g* (P40) have been used in Arabidopsis [21,22,23,24,49,50,51]. By determined by electron microscopy (EM), 70% of plant EVs in the P100 fraction had a size of 30–100 nm in diameter, while only 59% of plant EVs in the P40 fraction were observed to have similar diameters [21]. The recent study further centrifugated the supernatant of the P40 fraction at 100,000× *g* to collect the P100-40 fraction [22]. Most EVs in P100-40 fraction (82%) were between 30 and 100 nm in diameter and contained large amounts of TET8-positive EVs [21]. Thus, centrifugation at 100,000× *g* can result in higher EV yields and higher efficiency in separating plant small EVs. Plant EVs in the P100 fraction can be further purified by gradient centrifugation. This method uses sucrose and iodixanol as classical media [21,22]. One plant EV subtype, TET8-positive EV, is accumulated in the iodixanol fraction with an average density of 1.08 g/mL, similar to the density of animal exosomes [21]. Although high-quality EVs can be obtained by gradient centrifugation, centrifugation takes a long time, generally more than 16 h. Immunoaffinity isolation can capture the specific EV subtype from heterogeneous vesicle groups within a short period [52]. For example, TET8-positive EVs have been successfully isolated by agarose beads conjugated with TET8 antibodies and can be directly used for further analysis [21,22]. Thus, immunoaffinity isolation is a powerful method to capture the specific plant EV subtype containing specific membrane proteins [4,53].

## 3. Isolation and Preparation Techniques for PENVs and APNVs

Unlike plant EVs isolated from apoplast washing fluid, PENVs are isolated from homogenized plant materials (tissue lysis method) [41,42,44] (Figure 1). This method destroys plant tissues and damages cells, which leads to combining membrane structures from organelles or plasma membranes with EVs [43]. For isolating PENVs, the freshly obtained plant juice undergoes a series of centrifugations: the first centrifugation at low speed (1000× *g*) removes large fibers and dead cells; the subsequent higher centrifugations (e.g., 5000× *g* and 10,000× *g*) remove cellular debris and large vesicles; finally, PENVs are precipitated by centrifugation at high speeds of 100,000–120,000× *g* [40,43,54]. PENVs obtained by differential centrifugation have a high yield and can be produced on a large scale in the laboratory. However, they are often mixed with nucleic acids, protein agglomerates, and other intercellular components [40,43,54]. The density gradient ultracentrifugation is the gold standard approach for further purification of crude extracts of PENVs. Using differential centrifugation and density gradient ultracentrifugation (layer-wise sucrose gradient), PENVs have been successfully isolated from various plants such as ginseng [55,56,57,58,59], ginger [40,60,61,62,63,64,65,66], cabbage [67], broccoli [54], bitter melon [68], grapes [43], grapefruit [38,46,69,70], lemon [71], orange [72], blueberry [73], coconut [74], carrot [75], apple [76,77], and citrus [35,42]. Currently, the gold standard in separating and purifying PENVs from plant juice is differential centrifugation followed by gradient ultracentrifugation [41,42,44].

Other techniques, such as ultrafiltration and size-exclusive chromatography, have been developed to isolate PENVs. Ultrafiltration collects vesicles based on the buoyant size of the particles [78]. For example, blueberry-derived PENVs were obtained by ultrafiltration of 0.45 μm-filtered juice using a 10,000 MWCO membrane [73]. Although ultrafiltration is simple, it cannot remove contaminants larger than the membrane intercepts, and the contaminants can clog the membrane and reduce filtration efficiency. Size-exclusive chromatography separates vesicles based on weight and molecule size, which is widely used to purify PENVs obtained by differential centrifugation. By using this method, obtained vesicles maintain high integrity and biological activity. For example, size-exclusive chromatography further separates two groups of vesicles, small vesicles smaller than 50 nm and large vesicles larger than 150 nm, from orange-derived PENVs [72]. In addition, the combination of size-exclusive chromatography and ultrafiltration obtained a relatively structurally homogeneous population of cabbage-derived PENVs [67]. Electrophoresis can further remove proteins and RNA outside of PENVs. This technique, combined with a 300 kDa cut-off dialysis bag, was used for isolating lemon juice PENVs that had a similar size and numbers to those separated by ultracentrifugation [79].

The membrane of PENVs has high stability and contains certain lipids that can bind to specific receptors in target tissues, making it possible for these lipids to be assembled into artificial vesicles, APNVs [46] (Figure 1). To assemble APNVs, the Bligh and Dyer method is widely practiced for lipid extraction from PENVs [38,60,64,65]. In this method, the hydrophilic and hydrophobic organic compounds of PENVs are separated by MeOH: CHCl_3_ (2:1, *v*/*v*) and CHCl_3_:ddH_2_O. Total lipids in the hydrophobic organic phase are obtained by drying after heating (60 °C) under nitrogen. After a vacuum pump removes residual chloroform, the dried lipids are immediately suspended by a solution with miRNAs or siRNAs. Afterward, the solution is performed bath sonication and passed through a high-pressure homogenizer (membrane filter), such as a liposomes extruder, to collect homogeneously sized APNVs. The above protocol is a standard method for assembling APNVs currently successfully obtained from grapefruit and ginger [38,61,64,70]. Moreover, APNVs can be stored at 4 °C for up to 25 days without changing physical properties, and this stability is important for drug or siRNA delivery [64]. Therefore, PENV-derived lipids can be packaged with various active ingredients to prepare APNVs, which have promising applications in the development of drug therapies.

## 4. The Characteristics and Identification of PENVs 

Like mammalian EVs, particle size distribution, concentration, and morphology are used to identify the physical characteristics of PENVs. Traditional methods used to characterize particle size include flow cytometry, nanoparticle tracking analysis (NTA), dynamic light scattering (DLS), scanning electron microscopy (SEM), and transmission electron microscopy (TEM) [80].

Without fluorescent labeling, flow cytometry’s lower particle size limit is about 500 nm [81], which is larger than many vesicles, such as exosomes (generally 50–150 nm in diameter) [1,53]. Flow cytometry can efficiently detect vesicles labeled with antibodies that recognize exosome surface-specific markers. NTA can quickly measure particle size and concentration of nanoparticles in the range of 10–2000 nm. DLS is an alternative technique for measuring particle size from 1 nm to 6 μm. Compared with NTA, DLS can only detect samples with higher concentrations. In addition, DLS is unsuitable for measuring complex particle samples of different sizes and cannot measure the concentration of particles [82,83]. By NTA or DLS analysis, grape-derived PENVs showed an average diameter of ~380.5 nm, and the purified ginseng-derived PENVs showed an average diameter of ~344.8 nm [43,59]. Ginger-derived PENVs are smaller, with an average diameter of ~188.5 nm [65]. Like plant EVs, PENVs derived from single plant tissues or organs may contain several particle subtypes. For example, PENVs isolated from citrus fruit juice showed two distinct peaks ranging from 75 to 155 nm and 235 to 245 nm [84]. Electron microscopy (EM) can visually see the morphology and size of particles. Like plant EVs, most PENVs are spherical, but sample fixation and dehydration result in cup-shaped morphology observed by SEM and TEM [85]. Cryo-EM avoids dehydration and chemical fixation, so EVs and PENVs under cryo-EM often exhibit spherical morphology [86]. Although EM can directly observe vesicles, long-time sample pretreatment and preparation are required [85]. Thus, EM is unsuitable for large and rapid measurements of vesicles.

## 5. The Molecular Composition of PENVs

The biochemical composition of PENVs overlaps with those identified for plant and mammalian EVs, such as lipids, proteins, and nucleic acids that regulate the physiological processes of target cells [35,42]. In addition to being used as a transport vesicle to load exogenous active molecules, PENVs contain functional biomolecules that can also have clinical therapeutic effects [42,44]. Despite many similarities between plant EVs and PENVs, they show some differences in lipid composition. The recent lipidomic analysis of Arabidopsis EVs showed a high abundance of three groups of lipids: sphingolipids (~46%), phospholipids (~21%), and sterols (~20%) [87]. The sphingolipids in Arabidopsis leaf EVs are nearly pure glycosyl–inositol–phospho–ceramides (GIPCs) [87]. The lipid compositions of PENVs are dependent on their source plants. PENVs contain fewer sphingolipids than plant EVs; most lipids are glycerolipids and phospholipids [36,88]. PENVs from orange juice contained phosphatidyl-ethanolamine (PE) (~40%), phosphatidylcholine (PC) (~25%), phosphatidylinositol (PI) (~12%), and phosphatidic acid (PA) (~5%), which was similar to that of grapefruit-derived PENVs [38,72]. Lipidomic data indicate that grape-derived PENVs are enriched with PA (~53.2%), PE (~26.1%), PC (~9.0%), and PI (~7.3%) [43]. Three major lipids in ginger-derived PENVs are PA (~42–47%), digalactosyl–diacyl–glycerol (DGDG) (~15–27%), and mono-galactosyl–diacyl-glycerol (MGDG) (~19–30%) [61,64,65]. PA in PENVs targets and stimulates the mammalian rapamycin target (mTOR), which is responsible for mammalian cell growth, proliferation, and recovery [89]. PA also enhances the accumulation and duration of PENVs in the gut [37]. Unlike most PENVs, enriched PC, and glycerophosphate, the majority of the lipids in ginseng-derived PENVs were digalactosyl–monoacyl–glycerol (DGMG, 59.4%), PE (16.8%), and ceramide (13.8%) [59]. Ceramide is only detected in ginseng-derived PENVs and may play an important role in macrophage polarization via mice’s toll-like receptor 4 (TLR4) activation [59]. Ceramide is enriched in animal exosomes and is one of the lipids critical for exosome biogenesis [90]. Whether ceramide is involved in plant EVs or PENVs biogenesis needs further study.

Proteomic analyses in plant EVs consistently identify proteins related to membrane trafficking, iron transport, defense, and reactive oxygen species (ROS) signaling [22,24]. Interestingly, approximately 59% of plant EV proteins are present in the early endosome (EE)/late endosome (LE) proteome, indicating that plant EVs are derived from endocytic trafficking [24]. The protein composition of EVs derived from *B. cinerea*-infected Arabidopsis leaves was previously studied using mass spectrometry [22]. Among the identified proteins, 28.24% were stress response proteins, and 14.88% were biotic stimulus response proteins [22]. The defense-related protein in EVs may contribute to plant defense against pathogens by trafficking these proteins to neighboring plant or pathogen cells. In addition, plant EVs contain cell wall-related enzymes, such as hydrolases, that might facilitate EV pass through the cell wall [91,92]. The other family widely identified in EVs is annexins, which are crucial in the biogenesis of mammalian EVs and for sorting the small RNAs into EVs [4,53]. In plants, annexins are found in EVs from Arabidopsis and sunflower seeds [22,25] and in PENVs from four citrus species, *C. sinensis*, *C. limon*, *C. paradisi*, and *C. aurantium* [93]. In PENVs from citrus, serval high-level proteins have been detected, such as patellin-3-like, clathrin heavy chain, heat shock proteins (HSPs), 14-3-3 protein, aquaporin, and glyceraldehyde-3-phosphate dehydrogenase [93]. The orthologues of these proteins have previously been reported in mammalian EVs. Until recently, the common and specific proteins in different PENVs were not clear, and the function of these proteins in biological and pharmacological activities is required to understand future studies.

Many studies showed that mammalian EVs are loaded with RNAs, such as small RNAs, fragmented and intact mRNAs, ribosomal RNAs (rRNAs), and long non-coding RNAs (lncRNAs) [94]. In plants, small RNA profiling revealed serval micro RNAs (miRNA), trans-acting small RNAs, and intergenic region-derived small RNAs specifically enriched in EVs, suggesting the selective sorting of small RNAs into EVs [22,23]. Further study showed some RNA binding proteins, Argonaute 1 (AGO1), RNA helicases (RHs), and annexins, contribute to small RNA loading and/or stabilization in EVs [22]. Similarly, specific small RNAs are enriched in strawberry-derived PENVs, and small RNA profiling in strawberry-derived PENVs differed from the whole strawberry juice [95]. The RNA content of PENVs in different plants vary from species to species. While they contain the same amounts of PENVs, grape and grapefruit PENVs contain much fewer RNAs than those isolated from ginger or carrot root PENVs [75]. The grape PENVs contain miRNAs enriched for the miR169 family, which share the sequence similarity in the seed region with two human miRNAs, has-miR-4480 and has-miR-4662a-5p [75]. Next-generation sequencing analysis of ginger-derived PENVs identified 109 mature miRNAs [37]. Some of them are stable in the gut, as ginger miR319a-3p still exhibited high levels over a 6 h feed period [37]. Some miRNAs inside the lemon-derived PENVs also showed stability in the gut, whose relative concentrations were increased after salivary digestion [96]. There are few reports of selective RNA sorting mechanisms in PENVs and the application of specific RNA in therapy.

## 6. Applications of PENVs and APNVs as siRNA Delivery Systems for Therapies

The lipid layer protects RNAs and other bioactive cargos in EVs in order to avoid degradation in the extracellular environment. Numerous studies showed EV-mediated functional transfer of miRNAs with a broad range of downstream effects in mammalian cells [8,10]. EVs also mediate cross-boundary small RNA trafficking between different species to mediate cross-species or cross-kingdom RNAi [30,97]. For example, the parasite nematode *Heligmosomoides polygyrus* derived-EVs transport miRNAs into mouse gut epithelial cells to modulate innate host immunity [98]. In plants, EV-mediated transport is the major pathway for the cross-kingdom trafficking of small RNA into fungal cells, which contributes to plant immunity [23].

**Figure 2:**
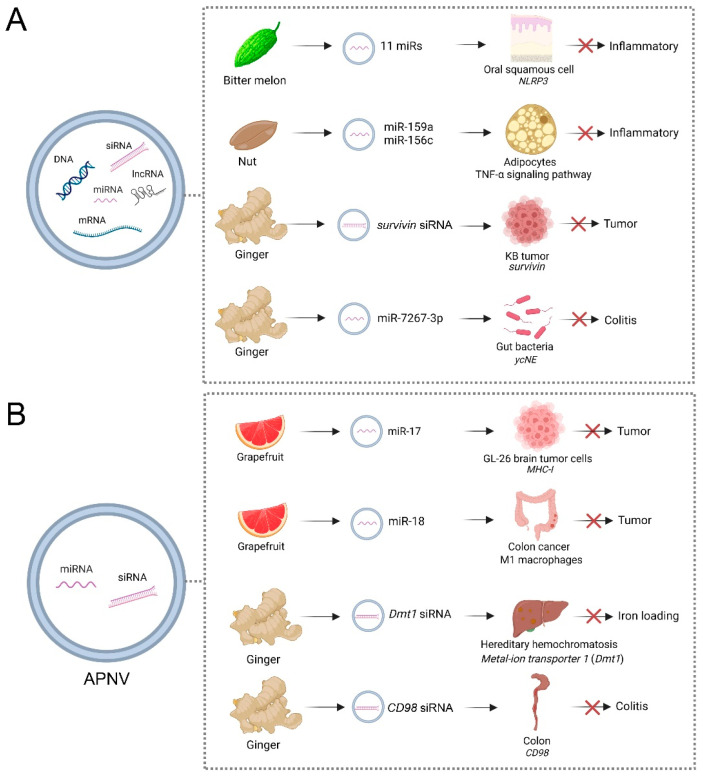
Applications of artificial plant exosome-like nanovesicles (PENVs) (**A**) and PENV-derived nano-vectors (APNVs) (**B**) in biotherapy for different animal diseases. PENVs and APNVs treat animal diseases by directly delivering functional miRNAs into animal cells. The effects of PENVs on the microbiome also can be used to target specific host processes to mitigate disease. Reference in (**A**): Bitter melon [68], Nut [99], Ginger [63,64]; (**B**): Grapefruit [38,70] and Ginger [61,64]. This figure was created with BioRender.com.

Cross-kingdom RNA transport by EVs paves the way for new applications of PENVs to regulate mammalian targets by plant small RNAs (Figure 2A). A recent study showed that bitter melon-derived PENVs exhibited anti-oral squamous cell carcinoma activities by enhancing the therapeutic effects of 5-Fluorouracil (5-FU) [68]. Fluoropyrimidine 5-fluorouracil (5-FU) is an antimetabolite widely used to treat cancer. However, activating the NOD-like receptor family pyrin domain containing 3 (NLRP3) inflammation induces oral squamous cell carcinoma resistance to 5-FU. The intrinsic anti-inflammatory functions caused by 11 miRNAs in PENVs downregulate the expression of *NOD-like receptor family pyrin domain containing* (*NLRP3*) [68]. After being injected peritumorally with 50 mg/kg 5-FU, PENVs + 5-FU, the tumors in the PENVs + 5-FU treatment group were significantly smaller than those in the 5-FU group and the PENVs group, indicating bitter melon-derived PENVs enhanced the cytotoxic effect and drug resistance of 5-FU in vivo [68]. In nut (*Juglans californica*)-derived PENVs, two conserved plant microRNAs, miR159a and miR156c, have been identified [99]. Nut miR159a and miR156c downregulated mice *TNF receptor superfamily member 1a* (*Tnfrsf1a*) transcript and reduced the level of pro-inflammatory cytokine-like TNF-α in adipocytes [99]. Compared with high-fat diet (HFD, 60% kcal from fat for 16 weeks) treatment, HFD + PENVs group showed reduced TNF-α mRNA levels and inflammation of visceral white adipose tissue in mice, indicating nut miRs have a potential role in treating inflammatory-associated metabolic diseases [99]. Ginger-derived PENVs showed similar size, density, and morphology to mammalian-derived exosomes [63]. By loading therapeutic siRNAs and fusing with folic acid (FA), ginger-derived PENVs were shown to deliver survivin (also known as *BIRC5*) siRNA to KB cancer cells by IV administration [63]. Ginger-derived PENVs had similar gene knockdown efficacy with transfection, and they effectively inhibited tumor growth, revealing the potential of PENVs to deliver siRNA [63]. Compared with controls, injection with 0.1 pmole PENVs/0.5 nmole siSurvivin-RNA per mouse (1 dose every 2 days; total 6 doses) showed suppressed tumor growth and no significant body weight changes, indicating ginger-derived PENVs as a delivery vector for therapeutic siRNAs with no gross toxicity [63]. In addition to being absorbed by the gut, PENV-carried miRNAs are absorbed by the gut microbiota, which alters microbiome composition and host physiology [37]. For example, ginger-derived PENVs carrying mdo-miR7267-3p are preferentially taken up by *Lactobacillus rhamnosus* (LGG), affecting the yields of LGG monooxygenase and ycnE, as well as increasing indole-3-carboxaldehyde, which affects IL-22 production [37]. By incubating 1 mg PKH26-labeled ginger-derived PENVs with 1 × 10^7^ LGG cells, the *ycnE* gene and protein were downregulated, suggesting PENVs affect gene expression and protein production in LGG [37]. Thus, the functions of ginger-derived PENV-RNAs alleviate mouse colitis through IL-22-dependent mechanisms. These findings reveal that the effects of PENVs on the microbiome can be used to target specific host processes to mitigate disease.

Lipids of PENVs assembled into APNVs were shown to be useful for delivering siRNAs into mammalian cells (Figure 2B). Grapefruit-derived APNVs mixed with polyethyleneimine effectively deliver miRNA-17 intranasally to the brain and are selectively taken up by folate receptor-positive GL-26 brain tumor cells [38]. Compared to controls, 20 nmol APNVs were administered intranasally, which can prolong survival in tumor-bearing mice, indicating this delivery had a therapeutic effect [38]. Administered through the nasal cavity, grapefruit-derived APNVs can reach the brain through the blood–brain barrier, and the miR17 they carry can also enter the brain to target genes [38]. Grapefruit-derived APNV carrying miR-18a can induce M1 macrophages to inhibit liver metastasis of colon cancer by directly targeting 3′UTR of IRF2 [70]. Compared with controls, tail veil injection with 200 nM PENVs/2 nM miR-18a per mouse (treated 3 times per week for 2 weeks) significantly reduced the number and size of tumor nodules in the livers and significantly prolonged mice survival [70]. Ginger siRNA-loaded APNVs can inhibit *divalent metal-ion transporter 1* (*Dmt1*)’s expression in intestinal epithelial cells from attenuating iron loading in a mouse model of hereditary hemochromatosis [61]. In this study, folic acid (FA) was infused with APNVs to improve its ability to target and integrate into the duodenum via proton-coupled folate transporters. Through treatment with 3.75 nmol *Dmt1*-siRNA -loaded -FA-GDLVs in mice, the expression of *Dmt1* was significantly reduced, causing a decrease in ferritin, TSAT, and non-heme Fe levels in organs such as the liver, kidney, pancreas, and heart [61]. In addition, APNVs carried siRNAs have effects on the digestive system. A recent study demonstrated that ginger-derived APNVs containing siRNA-CD98 could prevent and treat gastrointestinal diseases caused by inflammation [64]. These APNVs effectively target specific colonic tissue, reducing *CD98* expression to control homeostasis in the gut [64]. Through oral administration, APNV-sRNA is absorbed in the intestine to regulate intestinal flora and inflammatory factors. Ginger-derived APNVs exhibit high biocompatibility compared to liposomal preparations and less toxicity to macrophages and colon-26 cells [64]. Oral administration of ginger-derived APNVs containing 3.3 nmol siRNA-CD98 targets the ileum and colon 12 h after administration, specifically decreasing *CD98* expression. In addition, siRNA-CD98 delivered via ginger-derived APNVs is approximately 10,000 times more efficient than naked siRNA-*CD98*, indicating APNVs are a novel and effective delivery of siRNA drugs for treating ulcerative colitis [64]. It is generally accepted that APNVs changed the current paradigm of siRNA delivery beyond artificial synthesis nanoparticles using naturally derived nanocarriers from edible plants.

## 7. Conclusions and Future Outlooks

In recent years, EV research has developed rapidly, from basic biological research to clinical applications. In mammals, EVs can reach distant organs, releasing their functional cargo in recipient cells to medicate cell-to-cell communications [1,10]. Emerging evidence showed that plant EVs are important in transporting cross-kingdom small RNA from hosts into interacting microbes in order to silence virulence-related genes [20,28,30]. Parasites also use this natural cell-to-cell communication pathway to communicate with mammalian host cells [98]. Therefore, EVs mediate small RNA communication cross-kingdom/cross-species, showing potential in treating human diseases. In addition, EVs have the potential to diagnose and treat diseases (including cancer, neurological diseases, and cardiovascular disease) and to be novel drug delivery systems [100].

According to studies in animal models, PENVs have the potential to deliver small-molecule drugs, such as small RNAs, through oral, intravenous, nasal, and transdermal administration. Due to the natural origin of plants, PENVs have several obvious advantages as therapeutic drugs to combat inflammatory diseases. Compared with artificial vesicles, such as synthetic nanoparticles and liposomes, PENVs have no detectable toxicity, low immunogenicity, better pH-dependent drug release properties, fewer side effects, and environmental friendliness [35,36]. PENVs are highly stable in extremely acidic environments in the stomach and in the gut’s highly active proteolytic enzyme environment [101]. A recent study showed PENVs deepen the penetration depth of the drug on the skin surface and in the intestine, and the amount of medicine absorbed by the cortex is greatly increased [102]. In addition, the surface of PENVs can be easily modified to improve their targeting or delivery ability. For example, grapefruit-derived EVs injected intravenously into pregnant mice do not cross the placental barrier [46]. Based on the above advantages, PENVs, and their derivatives, have excellent application prospects and strong competitiveness in future clinical applications and preventive health care.

Although PENVs of different plant origins have been used as biotherapeutics or drug carriers to treat various human diseases, the study of PENVs is still in its infancy. Some key questions must be solved to apply them better in order to maintain human health: (1) Current differential ultracentrifugation for extracting and separating PENVs is unsuitable for large-scale production. How can PENV extraction and purification processes be standardized to make it an effective tool for biological therapeutics? (2) The currently extracted PENVs are highly heterogeneous, containing multiple vesicle types, and of unknown origin in plant cells. What are the mechanisms and roles of different PENV vesicle types in therapeutic processes? (3) There is currently a lack of standards for common or specific components of PENVs. (4) Do unknown bioactive ingredients in plants present some biosafety and toxicity-related challenges in applying PENVs or APNVs? (4) How can the stability of PENVs and APNVs be improved to advance their biotherapeutic applications? (5) Because PENVs and APNVs do not come from mammalian cells, they may have poor targeting ability in some targeted tissues. How can it be improved? (6) How can the efficiency of PENV-siRNA and APNV-siRNA in silencing target genes in specific organs be increased? Although the above problems have yet to be answered, plant vesicles mediate cross-kingdom regulation between plants and the human body, are a worthy field for future study, and have shown strong potential as a next-generation therapeutic strategy.

## Data Availability

Not applicable.
